# The Washington State Environmental Health Disparities Map: Development of a Community-Responsive Cumulative Impacts Assessment Tool

**DOI:** 10.3390/ijerph16224470

**Published:** 2019-11-13

**Authors:** Esther Min, Deric Gruen, Debolina Banerjee, Tina Echeverria, Lauren Freelander, Michael Schmeltz, Erik Saganić, Millie Piazza, Vanessa E. Galaviz, Michael Yost, Edmund Y.W. Seto

**Affiliations:** 1 Department of Environmental and Occupational Health Sciences, University of Washington, Seattle, WA 98195, USA; airion@uw.edu (M.Y.);; 2 Front and Centered, Seattle, WA 98122, USA; deric@frontandcentered.org; 3 Puget Sound Sage, Seattle, WA 98104, USA; debolina@pugetsoundsage.org; 4 Washington State Department of Health, Olympia, WA 98504, USA; tina.echeverria@doh.wa.gov (T.E.); lauren.freelander@doh.wa.gov (L.F.); 5 Department of Health Sciences, California State University, East Bay, Hayward, CA 94542, USA; michael.schmeltz@csueastbay.edu; 6 Puget Sound Clean Air Agency, Seattle, WA 98101, USA; ErikS@pscleanair.org; 7 Washington State Department of Ecology, Olympia, WA 98504, USA; millie.piazza@ecy.wa.gov; 8 Office of Environmental Health Hazard Assessment and Office of the Secretary, California Environmental Protection Agency, Sacramento, CA 95814, USA; vanessa.galaviz@calepa.ca.gov

**Keywords:** environmental justice, geospatial mapping, geographic information systems, community engaged, community driven research

## Abstract

Communities across Washington State have expressed the need for neighborhood-level information on the cumulative impact of environmental hazards and social conditions to illuminate disparities and address environmental justice issues. Many existing mapping tools have not explicitly integrated community voice and lived experience as an integral part of their development. The goals of this project were to create a new community–academic–government partnership to collect and summarize community concerns and to develop a publicly available mapping tool that ranks relative environmental health disparities for populations across Washington State. Using a community-driven framework, we developed the Washington Environmental Health Disparities Map, a cumulative environmental health impacts assessment tool. Nineteen regularly updated environmental and population indicators were integrated into the geospatial tool that allows for comparisons of the cumulative impacts between census tracts. This interactive map provides critical information for the public, agencies, policymakers, and community-based organizations to make informed decisions. The unique community–academic–government partnership and the community-driven framework can be used as a template for other environmental and social justice mapping endeavors.

## 1. Introduction

Institutional environmental justice (EJ) initiatives have focused on promoting environmental equity and social justice through the meaningful involvement of impacted communities and equitable distribution of the environmental burdens [1]. These efforts are framed as a response to procedural and distributive injustices that have contributed to disparities in exposures to environmental hazards and threaten the health and well-being of communities of color and low-income populations in the United States. Specifically, procedural justice addresses the historical imbalances in privilege, power, and representation that effectively exclude these populations from influencing the multitude of environmental decisions that impact communities [1,2,3,4]. Distributive justice addresses the inequitable distribution of environmental burdens across communities [1,4]. In Washington State, there is a need to identify communities where health disparities are likely to occur because of environmental injustices.

Identifying communities with high pollution burden and who are vulnerable to pollution’s effects is important for advancing environmental justice. Understanding the unequal distribution of environmental hazards or risks is important for developing solutions to environmental health disparities. Cumulative environmental impact assessment tools can help quantify the so-called “double jeopardy” issue—the combined and interactive exposure to environmental hazards and socioeconomic stressors that contribute to environmental health disparities [5,6]. This additional consideration of population vulnerability is typically not fully appreciated within a traditional risk assessment methodology. Methods for cumulative impact assessment are emerging [6,7,8], often implemented in the form of screening tools to help identify the likelihood of the occurrence of environmental health disparities [9].

Existing tools, such as the US Environmental Protection Agency’s (EPA) Environmental Justice Screening and Mapping Tool (EJSCREEN, EPA, Washington, D.C., USA) and the California Office of Environmental Health Hazard Assessment’s (OEHHA, Sacramento, CA, USA) California Communities Environmental Health Screening Tool (CalEnviroScreen, Sacramento, CA, USA), have developed methodologies to identify communities disproportionately impacted on by pollution burden while integrating population characteristics data to account for intrinsic and extrinsic vulnerabilities. Fundamental differences exist between the EJSCREEN and CalEnviroScreen frameworks. The EJSCREEN assesses excess environmental risk of exposure to environmental hazards and burden on communities by comparing populations in census blocks to other census blocks across the nation. It also provides multiple indices based on individual environmental risk factors rather than creating a single composite score that integrates multiple risk factors. In contrast, CalEnviroScreen assesses the cumulative environmental impacts of various risks to communities at the census tract level in California. 

These mapping tools are fundamental to environmental justice, as they illuminate the disparities in environmental health conditions across populations. However, strategies to promote distributive justice need more inclusive and systematic ways to actively engage impacted communities for environmental decisions. In California, before the creation of CalEnviroScreen, the OEHHA convened a research team and science advisory board to develop new methods for cumulative impact assessment and precautionary approaches. The CalEnviroScreen, as a tool for systematically quantifying EJ cumulative impacts, was the result of policy implemented by people of color from disadvantaged communities who worked with EJ leaders to write bills which were passed that mandated the integration of EJ into policy and the development of CalEnviroScreen. While there was community and EJ leadership advocating for greater consideration of EJ and tools, the methodology for CalEnviroScreen was developed from an agency-driven, top-down approach, and it is unclear what role community residents and organizations played in the selection of environmental risks and how they would be combined to quantify cumulative risk. The development of tools, like CalEnviroScreen in California and EJSCREEN at the national level, have implications for procedural justice (i.e., how communities with EJ considerations are identified, and who gets a seat at the table when working on EJ policy issues) and distributive justice (i.e., determining which communities are prioritized for corrective/restorative actions, and which communities bear environmental burdens). Only a few articles have been published studying the relationship between existing tools to known disparities related to socioeconomic status, race/ethnic groups, and health outcomes [10,11,12].

Recent work has demonstrated that a community-driven framework may be used to develop EJ tools [13]. University of Maryland researchers, stakeholders, and residents representing various EJ issues in Prince George’s County in the state of Maryland (MD), worked collaboratively to build the MD EJSCREEN, based on the USEPA’s EJSCREEN framework [13]. The MD EJSCREEN integrates national, statewide, and regional specific indicators to represent the cumulative environmental impact of risk for the state of Maryland through community feedback and engagement early in the project. However, the authors limited the outreach and engagement efforts to a specific county of Maryland, Prince George’s County. Although Prince George’s County represented a racially diverse population affected by environmental injustices, the authors did not state efforts to engage more communities outside of this county [13].

Independently, over a two-year period, a work group in Washington State collaborated to develop a new cumulative impacts mapping tool which was based on a community-engaged process. This work built upon the lessons and expertise of preceding mapping projects with the intent to create the first cumulative impact of environmental hazards and resulting disparities across the state. 

The goals of the Washington Environmental Health Disparities Map project were to create a community–academic–government partnership to develop an EJ map and to create a map that ranks relative environmental health disparities of communities in Washington State using a community-driven framework. The goal of this paper was to summarize our process, the methodological framework for integrating environmental and population indicators into cumulative impact rankings, environmental justice findings for the state based on the first version of the mapping tool, and the policy implications of the tool.

## 2. Materials and Methods 

In early 2017, the Washington EJ Mapping Work Group was initiated by Front and Centered, an EJ coalition of organizations rooted in communities of color, in partnership with Puget Sound Sage, University of Washington Department of Environmental and Occupational Health Sciences (DEOHS), the Washington State Department of Health (DOH) Washington Tracking Network (WTN) program, the state Department of Ecology (ECY), and the Puget Sound Clean Air Agency (PSCAA), a regional air quality management agency.

Front and Centered coordinated the work group meetings, led community engagement efforts, provided feedback for the project, and monitored progress of the project. The DEOHS took the lead in the technical aspects of the project, conducted the literature review, developed models for datasets, and conducted sensitivity analysis for the map. The DOH WTN staff provided input on the methodology and data used for the tool in addition to staff time and resources required to integrate the map into their mapping platform. The ECY staff provided insight for the environmental data for Washington State and the potential application of other Washington State-specific data for the project. The PSCAA staff offered technical expertise for air quality data in addition to sharing experience mapping community vulnerability at a regional level in Washington State.

Monthly meetings were held between January 2017 and February 2019 to discuss the expectations of each partner, review the timeline and progress on the project, provide feedback on the content, and report back mapping-related activities to the rest of the work group. The goal of the Washington EJ Mapping Work Group was to oversee the development of the Washington Environmental Health Disparities Map through the community–academic–government partnership.

### 2.1. Community Listening Sessions 

In mid-2017, Front and Centered issued a request for proposals (RFPs) from community-based organizations across the state to host listening sessions. Eleven different community organizations hosted a series of community listening sessions to discuss the environmental health risk factors communities have faced [14]. Two questions were asked: (1) What kinds of pollution, if any, are impacting your life or work and that of your family and community? (2) What factors best show if your community is healthy or doing well compared to other communities?

Front and Centered developed a facilitator’s guide and accompanying materials, including a sign-in sheet with zip codes, a note-taking template, and a summary template for facilitators. Community leaders from host organizations facilitated the sessions, took individual notes, and summarized each of their meetings. 

**Figure 1:**
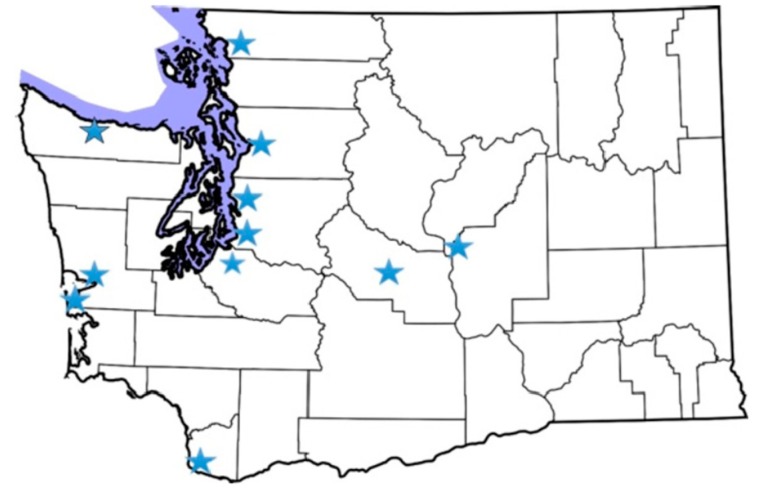
Stars noting the location of listening sessions held between July and November 2017.

Eleven two-hour community listening sessions were hosted between July and November 2017 with over 170 participants (Figure 1). The primary audience for engagement were communities across Washington who were identified through literature as disproportionately vulnerable to cumulative environmental burdens, particularly communities of color, households with lower incomes, immigrants and refugees, and linguistically isolated groups. 

The common themes identified in these listening sessions were used to inform the work group on indicator selection. Detailed results from the listening session are presented in a dedicated report [15].

### 2.2. EJ Mapping Symposium 

In February 2018, over fifty participants from research, government, and community-based organizations convened for a daylong work session to discuss potential indicators for the tool, methods for determining and illustrating the severity of environmental health disparities and the impact of climate change on environmental factors in communities across the state. A portion of the symposium included breakout sessions with participants discussing four key areas: population characteristics, environmental effects and exposures, climate impacts, and application of policy in practice. The discussion groups then came together to share summaries from each group discussion and propose new potential indicators of environmental health disparities, such as wealth inequality, concerns (such as accounting for undocumented and indigenous people), and the need for actionable data and information at the community level.

### 2.3. Literature Review and Indicator Selection

The DEOHS conducted a literature review for the potential data sources that could inform indicators identified through the series of community listening sessions and the EJ mapping symposium. Data sources were reviewed for statewide availability, reliability of the data source, and quality of data at the census tract level. At this time, other existing EJ mapping tools were reviewed for their methodology and inclusion of specific indicators.

From April to July 2018, the work group reviewed the secondary data and literature, and reached consensus for selection of specific indicators and methodology to create indicators if needed, and the cumulative impact framework to model, score, and rank environmental risks in Washington State. 

### 2.4. Draft Map Development

The DOH integrated the selected indicators and methodology into the Washington Tracking Network, a platform featuring publicly accessible data on more than 300 measures of environmental risks and public health. Based on feedback from the work group, the DEOHS developed a draft technical report specifying the methodology for each indicator [15]. Two different sensitivity analyses were conducted: Spearman’s correlation coefficients and principal component analysis. Principal component analysis (PCA) was conducted using the “prcomp” function in R (Version 3.6.0, The R Foundation for Statistical Computing, Vienna, Austria) and Rstudio (Version 1.2.1335, Boston, MA, USA) [13].

Once a draft map was created, the work group members hosted a webinar in September 2018 to share the findings in the draft report. Organizations that hosted the listening sessions, staff from government agencies such as OEHHA and ECY, stakeholders from partner organizations, and academic researchers in related fields were invited to the webinar. More than 90 people attended the webinar or listened to the webinar recording. The work group was able to gather participant feedback on ways to frame the environmental risks and environmental health disparities captured in the final map and interpret its findings.

### 2.5. Communication Planning

A subgroup consisting of communication experts in the work group met biweekly from October 2018 to January 2019. The subgroup outlined the communication goals in order to effectively and collaboratively launch the inaugural map. The subgroup also created a shared document to solicit feedback from one another and negotiate the description of the tool, roles of each, and background information on the project. The communication plan also included consistent terminology to be used by all partners and how to frame environmental risk, health impacts, and burden related to the mapping tool. At this time, work group partners named the tool “Washington Environmental Health Disparities Map”.

### 2.6. Launch of Washington Environmental Health Disparities Map

The work group partners worked together to release a press advisory to formally launch the tool in January 2019. In addition, Front and Centered hosted a Statewide Environmental Justice Summit to release the Washington Environmental Health Disparities Map to more than 200 community members and organizers.

## 3. Results

### 3.1. The Model

A review of the literature and methods for EJSCREEN and CalEnviroScreen suggested that the CalEnviroScreen model was better aligned with the goals of the work group. The CalEnviroScreen model focused on producing cumulative impact scoring across a variety of environmental hazards and population characteristics for communities in the state as opposed to evaluating risk based on individual hazards as provided in the EJSCREEN model. Therefore, similar to CalEnviroScreen, the inaugural version of the Washington Environmental Health Disparities Map was based on a model that integrates measures of environmental exposures, adverse environmental effects, sensitivities, and sociodemographic vulnerabilities together to create a single composite score [16]. The approach was based on scientific support—from existing research, risk assessment principles, and established risk scoring system—that vulnerability at an individual or community level modifies environmental risk for communities [17]. 

The Equation (1) used in this model was based on the established risk scoring [16,17]:

Risk = Threat × Vulnerability(1)

The Equation (2) was modified for our model accordingly:

Disparities Rank = Environmental Exposures and Effects × Sensitive Populations and Socioeconomic Factors(2)

Final Score = Pollution Burden Score × Population Characteristics Score(3)

The Pollution Burden score summarized the environmental risk factors and hazards in communities. It was calculated by modeling the pollution burden from the levels of certain pollutants that populations come into contact with and are exposed to directly. Threat also captured indicators that account for the damage to environmental quality, which can increase environmental risk factors for nearby communities.

The Population Characteristics score was represented by various biological and non-biological characteristics at a community level. Characteristics captured in this category were proxy metrics for population characteristics that represent vulnerability to environmental risk and may affect the susceptibility or resilience to pollution burden, including educational attainment and poverty. These characteristics modified the environmental risk.

### 3.2. The Indicators

The indicators in the map were assigned to one of the four categories: (a) Environmental Exposures (measurement of pollutants), (b) Environmental Effects (adverse environmental quality that may pose a risk to nearby communities), (c) Sensitive Populations (biological/intrinsic vulnerability in a community), and (d) Socioeconomic Factors (extrinsic vulnerabilities that modify resilience to environmental hazards). Data sources included US EPA, US Census Bureau, DOH, and ECY.

For each indicator, individual census tracts were assigned a decile score, based on rank-order of the raw values. The Environmental Exposures and Environmental Effects category were combined into the Pollution Burden score (maximum score of 10), based on the Equation (4):

(4) $$\begin{array}{c} \mathrm{Pollution}\;\mathrm{Burden}\;\mathrm{score}=\\ \frac{\mathrm{Avg}\;\mathrm{percentile}\;\mathrm{score}\;\mathrm{of}\;\mathrm{Environmental}\;\mathrm{Exposures}\;\mathrm{indicators}+0.5\times \mathrm{Avg}\;\mathrm{percentile}\;\mathrm{score}\;\mathrm{of}\;\mathrm{Environmental}\;\mathrm{Effects}\;\mathrm{indicators}}{2} \end{array}$$

Note that the percentile score for Environmental Effects Indicators is half weighted because of uncertainties in the extent to which proximity to hazardous sites and pollutant sources reflects exposures to individuals in the community. This follows a similar methodology used by CalEnviroScreen. The Sensitive Populations and Socioeconomic Factors categories were combined into the Population Characteristics score (maximum score of 10), based on the Equation (5):

(5) $$\begin{array}{c} \mathrm{Population}\;\mathrm{Characteristics}\;\mathrm{score}=\\ \frac{\mathrm{Avg}\;\mathrm{percentile}\;\mathrm{score}\;\mathrm{of}\;\mathrm{Sensitive}\;\mathrm{Populations}\;\mathrm{indicators}+\mathrm{Avg}\;\mathrm{percentile}\;\mathrm{score}\;\mathrm{of}\;\mathrm{Population}\;\mathrm{Characterstics}\;\mathrm{indicators}}{2} \end{array}$$

When displaying the final disparities rank, a decile ranking of 1–10 is subsequently used in the resulting map.

The indicators for each category are shown in Table 1.

### 3.3. Spearman’s Correlation between Indicators

In an effort to reduce duplicative indicators, Spearman’s correlation was used to determine the relationship among each indicator values included in the map (shown in Table 2).

Within the environmental exposure category, only diesel emission and PM_2.5_ were moderately correlated (*p* = 0.51). Proximity to Hazardous waste Treatment, Storage and Disposal Facilities (TSDF) facilities were moderately correlated with toxic releases from facilities and proximity to Superfund/NPL sites (*p* = 0.52 for both). Poverty (185% below federal poverty level) was highly correlated with education (*p* = 0.70) and moderately correlated with housing burden (*p* = 0.57). Linguistic isolation was highly correlated with race/ethnicity (*p* = 0.81). Transportation expense was negatively correlated with diesel emission (*p* = −0.78).

Based on the correlation coefficients, only linguistic isolation and race/ethnicity were found to be highly correlated indicators. Since each of these two indicators capture different vulnerabilities, both indicators were selected to remain in the final Washington Environmental Health Disparities Map (e.g., linguistic isolation captures those that may experience difficulty accessing environmental information in non-English material while race/ethnicity indicator captures minority populations).

### 3.4. Washington Environmental Health Disparities Map, Version 1.0

**Figure 2:**
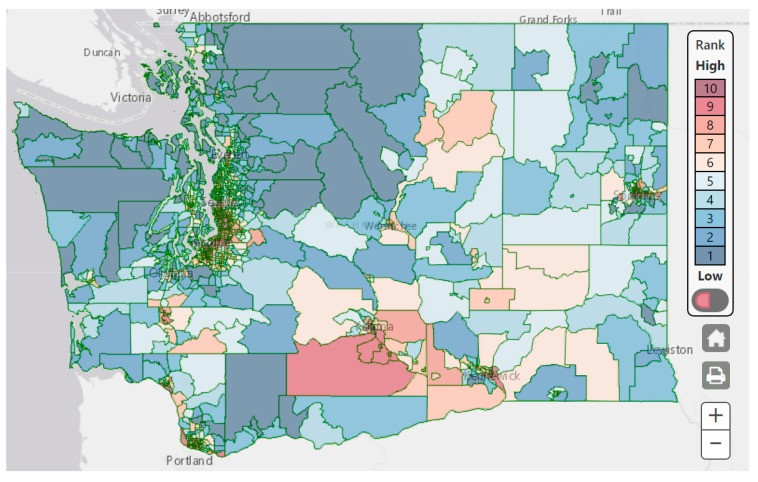
Screenshot of the final environmental health disparities (EHDs) ranking of the Washington Environmental Health Disparities Map version 1.0.

The underlying indicators (including descriptions of data sources, data methods, and links to download data) and cumulative risk results are accessible as a free, publicly available online mapping tool developed and maintained by DOH WTN [19]. The tool supports interactive zooming and panning, searching for specific locations, selection, and viewing of individual indicators and categories and overall risk. Based on the final score (Equation (3), the Washington Environmental Health Disparities Map depicts the final environmental health disparities (EHD) ranking from 1 to 10, with 10 indicating the highest cumulative impact due to the environmental risks and vulnerabilities. These rankings reflect the risk each census tract faces from pollution and vulnerabilities relative to other census tracts in Washington. A screenshot of the resulting map on the website is shown in Figure 2.

### 3.5. Principal Component Analysis (PCA)

**Figure 3:**
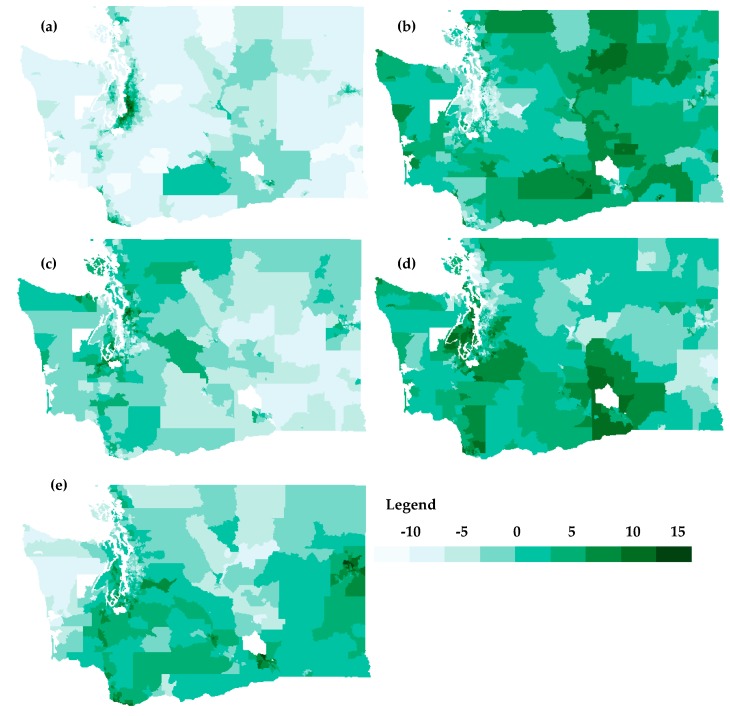
Map of components from the PCA: (**a**) urbanized areas (diesel emissions, PM_2.5_, people of color, linguistic isolation); (**b**) socioeconomic factor (poverty, low educational attainment); (**c**) traffic−related pollution (traffic density); (**d**) hazardous waste (proximity to hazardous waste, toxic releases from facilities); (**e**) Peri−urban/Superfund−related pollution (ozone, proximity to Superfund sites).

We used PCA to understand the groups of indicators that influence the final ranking. Rank−order of the raw values for each indicator were used in order to account for unit variability among indicators. Based on the results of the preliminary PCA analyses, low birth weight and cardiovascular disease data were excluded due to the fact that both factors did not have a strong weight in any of the main principal components. After examining the scree plot, five principal components were selected accounting for 66.26% of the variance. The components corresponded approximately to (1) pollution related to urbanized areas, (2) socioeconomic factors, (3) traffic−related pollution, (4) hazardous waste, and (5) peri−urban related pollution, with each accounting for 28.71%, 14.43%, 8.41%, 7.77%, and 6.95% of the variance, respectively (Figure 3). 

### 3.6. Race and Income

**Figure 4:**
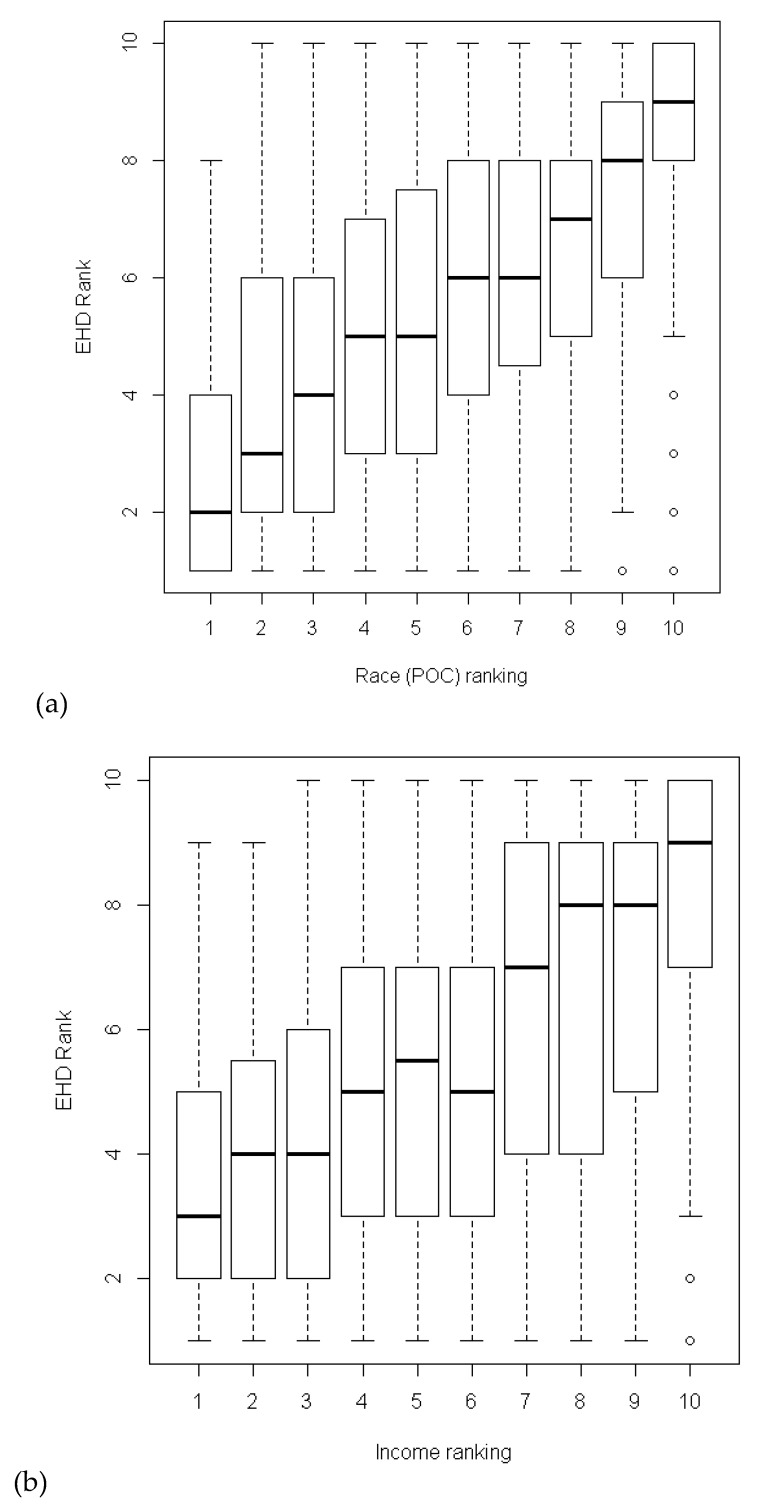
Distribution of the final EHD ranking: (**a**) by race/people of color (POC) indicator ranking and (**b**) income/poverty indicator ranking. The bar shows the median ranking for each group. The box shows the interquartile range.

The final EHD ranking, based on race and income, shows that census tracts with a higher proportion of people of color and a population living below 185 percent of the federal poverty level are more likely to experience higher environmental health disparities (Figure 4).

### 3.7. Communities Highly Impacted by Environmental Health Disparities (80th Percentile)

**Figure 5:**
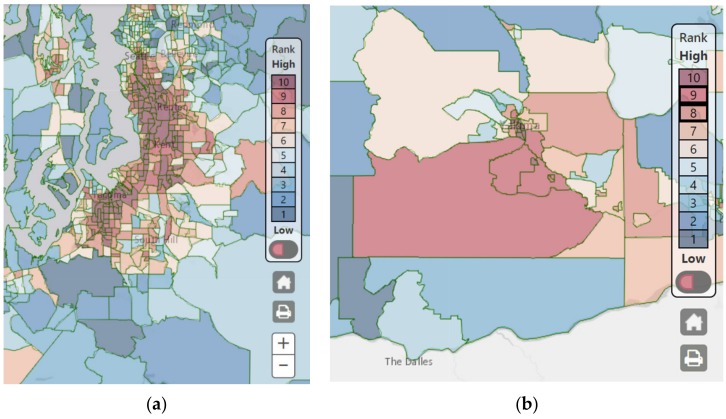
Screenshot of the final EHD ranking: (**a**) South Seattle, Kent, and Tacoma area; (**b**) the Yakima Valley region.

Approximately eight clusters were identified for areas ranked “9” or “10” or the top 20 percent (80th percentile) of highly impacted communities in both western and eastern Washington. These included urban areas such as South Seattle, Kent, Tacoma, Vancouver, and Spokane and rural areas such as Centralia, Longview, Yakima Valley, and the Tri−Cities. Environmental risk factors driving the final score to a “9” or a “10” varied depending on the region. For example, a cluster of census tracts in urbanized areas of South Seattle, Kent, and Tacoma were ranked in the top 20 percent (Figure 5a). Environmental health disparities in these tracts were influenced by diesel emission, traffic density, toxic release from facilities, proximity to Superfund/NPL sites, and housing burden that ranked “9” or “10” for these individual indicators. Rural census tracts in Yakima Valley were also ranked in the top 20 percent due to the indicators such as PM_2.5_, wastewater discharge, poor educational attainment, and transportation expense (Figure 5b). Both of these areas were similarly and highly impacted by linguistic isolation, people of color, poverty, and cardiovascular disease.

## 4. Discussion

To our knowledge, this is one of the first community−driven frameworks for mapping statewide environmental justice issues. The community–academic–government partnership for this project was relatively new, formed in an ad−hoc manner through initiation from Front and Centered. The project was made possible by leveraging existing resources within the partnering organizations, presenting potential challenges for a new, multifaceted partnership. However, explicit efforts were made to integrate procedural justice throughout the project. Negotiating and establishing goals of the project at the partnership formation became a solid foundation for the work group to succeed in this project. In addition, listening to the experiences of different communities at the early stages of the project, and being responsive to community input and direction were also critical for the project’s success. 

### 4.1. Findings

The Washington Environmental Health Disparities Map identifies communities most heavily impacted by the environmental risks and vulnerabilities. Results from the PCA demonstrate opportunities for more targeted priorities for different regions of the state. For example, Figure 3a shows diesel emissions may be most relevant for urbanized area, especially in communities of color. In contrast, Figure 3b indicates areas that are suffering from low socioeconomic status that may benefit from strategic public health programming. 

The final EHD ranking also suggests that people of color and poverty are likely to experience higher pollution and increased vulnerabilities to pollution’s effects [20,21]. The Washington Environmental Health Disparities Map framework captures race and income as two key populations that are affected more by environmental health risks [22,23]. 

### 4.2. Data and Methodological Needs

By working in partnership with state agencies with access to both public health and environmental monitoring data, we were able to identify data of high quality that are maintained and routinely updated. This was a strong requirement for our inclusion of specific indicators to help promote the sustainability of the tool and the ability to assess changing environmental health disparities as community conditions change over time. At the same time, this project identified data gaps and methodological needs that warrant more attention. For example, quantifying the prevalence of asthma or cardiovascular disease in each census tract can help identify communities that are more sensitive to pollution [24,25,26,27]. However, Washington State does not currently maintain an easy−to−access database to measure prevalence of these chronic conditions. Proxy measures such as emergency department utilization rates are under development but not yet available.

As another example, drinking water contaminants are difficult to model and measure. The participants in the community listening sessions emphasized the importance of safe, clean drinking water. While public water systems are required to report annual water quality data, private wells are not. This poses a challenge when modeling drinking water contaminants for Washington State, as approximately 15% of Washington residents (over 1 million people) rely on private wells for drinking water [28]. 

Many of the indicators in this map rely on national data sources. While nationwide data provide insight into environmental health burdens at the national level, these data may not capture the nuances that state−specific data would. Therefore, more research is required to model the state−specific data, such as a community’s proximity to state−specific cleanup sites in addition to the NPL sites. In addition, more effort to collect regional and statewide data sustainably is critical to improve maps such as the Washington Environmental Health Disparities Map. 

The cumulative environmental risk framework we used is critical in mapping environmental health disparities. This map intentionally does not model resilience or asset−based indicators contributing to environmental health. This map also does not model the overall burden on communities nor does it reflect the actual number of individuals affected by environmental burden. Further, the map does not model the positive or negative likelihood of an individual health outcome. However, the authors acknowledge the importance of a parallel asset−based map, as emphasized by communities during the listening sessions.

### 4.3. Potential Uses in Policy and Practice

A statewide mapping tool showing the cumulative impact of environmental risk can strengthen the ability of government agencies and policy makers to more systematically identify and quantify drivers of disparities in the pursuit of environmental justice [3]. Additionally, the Washington Environmental Health Disparities Map can be used assist in resource allocation and decision−making. This can be done through identifying and designating highly impacted communities to receive a proportion or the entirety of a resource, through scaling a resource investment proportional to the risk level, or through other strategies that direct investment. By focusing on highly impacted census tracts identified by the Washington Environmental Health Disparities Map, the state can direct investments, programs, and other resources to ensure environmental and public health equity. As an example, recently passed legislation, Washington Senate Bill (SB) 5116 (2019)—the state’s Clean Energy Policy—requires equitable distribution of energy and non−energy benefits and reduction of burdens to vulnerable populations and highly impacted communities through the use of cumulative impacts analysis [29].

Another potential use of the Washington Environmental Health Disparities Map is to improve public health through strategic and meaningful community engagement in census tracts with high cumulative impacts. Communities burdened with environmental health disparities may receive less attention from governmental agencies [30]. These communities may also face additional barriers to participation such as insufficient or sometimes exclusionary outreach and information dissemination by public entities, lack of resources and time to attend, language barriers, literacy differences, and health issues [31,32]. There is scientific evidence that community engagement reduces health disparities due to the presence of factors such as improved knowledge and self−efficiency [32,33]. Additional benefits from increased community engagement include reciprocal knowledge translation, improved community−stakeholder relationships, and improvements in the Washington Environmental Health Disparities Map as new concerns are identified and data sources are developed to respond to those concerns. 

Policies should also recognize tribal areas as highly impacted areas for environmental health disparities. Several tribal members and representatives from Native American organizations were engaged in the community listening sessions, and actively provided feedback through participation in the symposium, webinar, and emails. Washington State is home to 29 federally recognized Native American tribes and several out−of−state tribes with treaty or traditional territory within the state and is home to numerous tribal communities throughout the state. Policies and actions intended to address cumulative environmental impacts across the state should be developed in consultation with the affected tribal governments and communities.

### 4.4. Limitations

This map was developed based on a specific model for relative pollution burden and vulnerabilities. Models have inherent uncertainty associated in the methodology of the tool. There is no single way to accurately capture the level of uncertainty associated with the cumulative impacts of all communities. However, this map represents a widely accepted science−based approach to quantify the cumulative environmental risks. 

### 4.5. Future Research Directions

The work group intends to update the map as statewide data for additional indicators become available. Partners in the work group plan to explore additional indicators such as asthma, noise pollution, proximity to state−specific clean−up sites, and quality of surface water. Other potential indicators require more development, such as drinking water quality, the effects of inequality and the effects of the built environment. Additional analysis is being conducted to make decisions on health outcomes that may be affiliated with the environmental risk factors.

The 2017 listening sessions included eleven communities and did not fully cover all geographic regions or communities within Washington State. As a result, the work group plans to continue to include input from more communities in the future to address this limitation.

## 5. Conclusions

Understanding the cumulative impacts from the complex interaction between pollution and vulnerability can allow informed decision−making to improve public health and the environment. Using the cumulative impacts assessment approach, we developed the Washington Environmental Health Disparities Map. The Washington Environmental Health Disparities Map allows for cumulative impact comparison among census tracts and provides the public agencies, policymakers, and community−based organizations critical information on disparities with which they can make informed decisions. In addition, the community−driven framework for building the map can be used as a template for other EJ mapping efforts to capture the voices of community in the map.

## Figures and Tables

**Table 1:** List of 19 indicators for the Washington Environmental Health Disparities Map, version 1.0.

Category	Indicators	Indicator description	Data Source
Environmental exposure	Diesel emissions	Combined gridded emissions, reallocated to census tracts using area-weighted spatial interpolation	Washington State Department of Ecology Comprehensive Emissions Inventory (2014)
Environmental exposure	Ozone	Three-year mean concentration of daily maximum 8 hour rolling averaged ozone	AIRPACT (2009–2011) [18]
Environmental exposure	PM_2.5_	Three-year mean concentration of annual PM_2.5_	AIRPACT (2009–2011)
Environmental exposure	Toxic releases from facilities	Toxicity-weighted concentrations of chemical releases to air from facility emissions and off-site incineration	Risk Screening Environmental Indicators (RSEI) (2014–2016)
Environmental exposure	Traffic density	Percentage of population exposed to busy roadways within each census tract	Washington State Office of Financial Management and Washington State Department of Transportation (2017)
Environmental effects	Lead risk and exposure	Total number of houses and proportion of houses by year of construction	ACS 5 year estimates (2012–2016)
Environmental effects	Proximity to hazardous waste generators and facilities	Count of all commercial Hazardous waste Treatment, Storage and Disposal Facilities (TSDF) facilities within 5 km, divided by distance, presented as population weighted averages of blocks in each census tract	EJSCREEN (2017)
Environmental effects	Proximity to Superfund sites	Count of sites proposed and listed on the National Priorities List (NPL)	EJSCREEN (2017)
Environmental effects	Proximity to facilities with highly toxic substances	Count of RMP facilities within 5 km, divided by distance, presented as population-weighted averages of blocks in each census tract	EJSCREEN (2017)
Environmental effects	Wastewater discharge	Toxicity-weighted concentration in stream reach segments within 500 meters of a block centroid, divided by distance in meters, presented as the population-weighted average of blocks in each block group	EJSCREEN (2017)
Sensitive Populations	Cardiovascular disease	Mortality rate from cardiovascular diseases for 2012–2016 per 100,000 population	Washington State Department of Health Center for Health Statistics (2012–2016)
Sensitive Populations	Low birth weight infants	Number of live born singleton (one baby) infants born at term (at or above 37 completed weeks of gestation) with a birth weight of less than 2500 grams (about 5.5 lbs.)	Washington State Department of Health Center for Health Statistics (2012–2016)
Socioeconomic Factors	Low educational attainment	Percent of population over age 25 with less than a high school education	ACS 5 year estimates (2012–2016)
Socioeconomic Factors	Housing burden	Modeled percent of income spent on housing for a four-person household making the median household income	ACS 5 year estimates (2012–2016)
Socioeconomic Factors	Linguistic isolation	Percent of limited English-speaking households	ACS 5 year estimates (2012–2016)
Socioeconomic Factors	Poverty	Percent of the population living below 185 percent of the federal poverty level	ACS 5 year estimates (2012-2016)
Socioeconomic Factors	Race (people of color)	Sum of all race/ethnicity categories except White/Non-Hispanics, including Black, American Indian/Alaskan Native, Asian, Native Hawaiian other Pacific Islander, and two or more races	Washington State Office of Financial Management (2015)
Socioeconomic Factors	Transportation expense	Transportation costs based on percentage of income for the regional moderate household	Center for Neighborhood Technology (CNT) (2014–2015)
Socioeconomic Factors	Unemployment	Percent of the population over the age of 16 that is unemployed and eligible for the labor force	ACS 5-year estimates (2012–2016)

**Table 2:** Spearman’s correlation coefficient for the 19 indicators and the composite scores. Indicators that are moderately (coefficient between −0.8 and −0.5 or between +0.5 and +0.8) or highly correlated (coefficient below −0.8 or above 0.8) are shown in gray highlights. Categories of indicators are colored.

		Exposure	Ozone	PM 2.5	Diesel Emission	Toxic Release	Traffic																	
	Environmental Exposure	1																						
Environmental Exposure	Ozone	−0.11	1					Environmental Effects	Lead Risk	Superfund Sites	Hazardous Waste	Risk Management Plan	Wastewater Discharge											
	PM2.5	0.64	−0.16	1																				
	Diesel Emission	0.74	−0.40	0.51	1																			
	Toxic Release	0.54	−0.38	0.21	0.36	1																		
	Traffic	0.69	−0.21	0.23	0.49	0.14	1																	
	Environmental Effects	0.56	−0.16	0.58	0.46	0.44	0.19																	
Environmental Effects	Lead Risk	0.16	−0.11	0.22	0.23	0.12	0.01	0.51	1					Sensitive Population	Cardiovascular Disease	Low Birth Weight								
	Superfund Sites	0.52	0.04	0.39	0.37	0.38	0.21	0.67	0.16	1														
	Hazardous Waste	0.51	−0.11	0.36	0.31	0.52	0.21	0.60	0.02	0.52	1													
	Risk Management Plan	0.29	−0.12	0.48	0.31	0.14	0.04	0.67	0.27	0.22	0.25	1												
	Wastewater Discharge	0.19	−0.18	0.29	0.16	0.16	0.09	0.51	0.13	0.14	−0.02	0.31	1				Socioeconomic Factor	Low Educational Attainment	Linguistic Isolation	Poverty	Unemployment	Housing Burden	Race/Ethnicity	Transportation Expense
	Sensitive Population	0.15	0.06	0.22	0.08	0.01	0.07	0.20	0.20	0.16	0.08	0.10	0.06											
Sensitive Populations	Cardiovascular Disease	0.08	0.11	0.17	−0.02	−0.10	0.05	0.09	0.10	0.05	0.06	0.05	0.04	0.67	1									
	Low Birth Weight	0.13	−0.02	0.13	0.11	0.10	0.03	0.19	0.15	0.20	0.05	0.09	0.09	0.69	0.08	1								
	Socioeconomic Factors	0.17	−0.03	0.33	0.12	−0.05	0.13	0.24	0.22	0.10	0.09	0.27	0.06	0.38	0.38	0.14								
Socioeconomic Factors	Low Educational Attainment	0.08	0.02	0.29	−0.03	−0.07	0.06	0.17	0.22	−0.02	0.06	0.24	0.04	0.40	0.41	0.13	0.82							
	Linguistic Isolation	0.33	−0.27	0.40	0.36	0.17	0.24	0.24	0.05	0.12	0.25	0.29	0.04	0.12	0.11	0.05	0.63	0.49						
	Poverty	0.09	0.11	0.21	0.01	−0.14	0.08	0.23	0.34	0.11	−0.04	0.21	0.09	0.39	0.36	0.17	0.82	0.70	0.32					
	Unemployment	0.03	0.11	0.08	0.00	−0.11	0.00	0.04	0.08	0.03	0.00	0.03	−0.02	0.18	0.19	0.06	0.50	0.33	0.12	0.39				
	Housing Burden	0.34	−0.14	0.27	0.41	0.09	0.27	0.27	0.18	0.25	0.17	0.13	0.11	0.28	0.25	0.14	0.60	0.34	0.31	0.57	0.25			
	Race/Ethnicity	0.40	−0.34	0.47	0.43	0.26	0.25	0.34	0.09	0.24	0.37	0.31	0.04	0.19	0.15	0.12	0.61	0.43	0.81	0.32	0.15	0.37		
	Transportation Expense	−0.60	0.46	−0.38	−0.78	−0.47	−0.40	−0.34	−0.13	−0.33	−0.38	−0.10	−0.11	−0.04	0.05	−0.09	−0.04	0.10	−0.32	0.05	0.05	−0.45	−0.47	
	Final Ranking	0.71	−0.02	0.65	0.52	0.30	0.43	0.65	0.33	0.51	0.45	0.45	0.25	0.63	0.45	0.43	0.62	0.51	0.49	0.51	0.26	0.53	0.56	−0.37

Spearman’s correlation coefficient based on IBL ranking.
